# Understanding Mental Health and Cognitive Restructuring With Ecological Neuroscience

**DOI:** 10.3389/fpsyt.2021.697095

**Published:** 2021-06-18

**Authors:** James Crum

**Affiliations:** Institute of Cognitive Neuroscience, University College London, London, United Kingdom

**Keywords:** neuroimaging, functional near infrared spectroscopy, ecological validity, mental health, hyperscanning methods, psychotherapy, cognitive-behavioral therapy

## Abstract

Neuroimaging and neuropsychological methods have contributed much toward an understanding of the information processing systems of the human brain in the last few decades, but to what extent do cognitive neuroscientific findings represent and generalize to the inter- and intra-brain dynamics engaged in adapting to naturalistic situations? If it is not marked, and experimental designs lack ecological validity, then this stands to potentially impact the practical applications of a paradigm. In no other domain is this more important to acknowledge than in human clinical neuroimaging research, wherein reduced ecological validity could mean a loss in clinical utility. One way to improve the generalizability and representativeness of findings is to adopt a more “real-world” approach to the development and selection of experimental designs and neuroimaging techniques to investigate the clinically-relevant phenomena of interest. For example, some relatively recent developments to neuroimaging techniques such as functional near-infrared spectroscopy (fNIRS) make it possible to create experimental designs using naturalistic tasks that would otherwise not be possible within the confines of a conventional laboratory. Mental health, cognitive interventions, and the present challenges to investigating the brain during treatment are discussed, as well as how the ecological use of fNIRS might be helpful in bridging the explanatory gaps to understanding the cultivation of mental health.

## Mental Health and the Brain

Mental health has long been a central topic of investigation in clinical psychology and psychiatry [see (1), for review], but it also has steadily been a growing subject of interest of government, education, and business institutions, as well as of other academic fields such as those comprising cognitive neuroscience and, importantly, of society on the whole. This sometimes leads to differences in how mental health is conceptualized, but it is most broadly understood as a latent construct for which people have normative reasons to want to try to cultivate (2) and, critically, it is probably not simply the absence of having a diagnosed psychiatric disorder. An analog to this is the concept of physical fitness: Physical fitness means different things to different people, but most would agree that it is important to overall well-being to try do things to develop it; although these “things” vary widely in variety and efficacy, they are at least clearly antithetical to physical stagnation. Physical fitness, then, is also not simply the absence of having a physical disease. More broadly, both physical well-being and mental health contribute and, indeed, largely comprise one's overall well-being. However, it is worth noting that, from the perspective of cognitive neuroscience, these dimensions of physiological and psychological well-being are not categorically disparate: A particular sense of reductionism is widely adopted in which a material substrate—the brain—is a necessary condition by which mental phenomena can emerge, including mental illness. So, because the dualism of the Mind-Body Problem (3) is generally rejected, psychological well-being is equivalent to the same sense of physical well-being as is considered when discussing, for example, damage to the body from a broken bone, but with a more specific focus on the integrity of the organ whose neural systems make possible all the conscious experiences people typically consider—at a more common-sense level of explanation—as involving mental health (e.g., complex beliefs, desires, and emotions about the self, others, and world).

## A Shift in Perspective and Diagnostics

Fortunately, the many initiatives and movements away from mental health stigma have been exceedingly impactful, in that more people are beginning to discern that although psychopathological symptoms such as experiencing emotion dysregulation and engaging in maladaptive behavior might suggest a disordered mind, the presence of symptoms is not indicative of there being something fundamentally wrong with them—something inherently weak in their personhood—and, consequently, more people are reaching out for help [e.g., (4)]. At the same time, access to non-pharmacological forms of treatment to those seeking it have opened markedly: for instance, internet-based cognitive-behavioral therapy (CBT). Several reviews and meta-analyses have shown that internet-based CBT is effective for reducing mild to moderate psychopathological symptoms (5–12). This mounting shift of the general public in seeing mental health as something similar to physical well-being—as something toward which everyone can work to better their lives—is accompanied by a theoretical one in the clinical sciences regarding diagnostic classification. Namely, there has been a push for incorporating more dimensional features into the formal nosology in recent years, and for even adopting a predominately dimensional approach [see (13), for review]. In addition to the collection of psychometric data, computational and multifactorial approaches to psychopathology in cognitive neuroscientific research are becoming increasingly popular [see (14), for review] and powerful in their aim to improve the reliability and validity of diagnostic classification by providing important idiographic information about individuals that might better account for within-category heterogeneity and inform intervention methods (15). For example, neurocognitive “endophenotyping” is a more comprehensive approach to capturing disordered thinking, feeling, and behaving and not only improves knowledge on the nature of psychopathology but also stands to better inform clinicians' assessments [see, (16), for review]. So, having diminished mental health is not always the same thing as having a psychiatric disorder and, conversely, having a clinical diagnosis is not a requirement to engage in things that cultivate mental health; therefore, it is important that interventions such as CBT that teach strategies for everyday life are encouraged and available to everyone.

## The Principle of Cognitive Mediation

In general, interventions (e.g., psychiatric treatment, physical activity, diet, etc.,) are the instruments by which well-being is chiefly cultivated. Diseases (acquired or innate) and environmental events, as well as certain aspects of thinking, feeling, and behaving, can detract from people's well-being either directly or instrumentally. On some views, thinking (i.e., cognition) is central to understanding mental health, because it mediates the relationship between stressful environmental events and decreases in aspects of mental health, such as experiencing unhealthy, negative emotional distress [e.g., (17)]. Indeed, the role of cognition in emotion is one of the oldest subjects of discussion in psychology, tracing as far back as ancient Greek philosophers such as Epictetus in 108_AD_, and was a topic around which there was considerable debate in the 20th century [see (18), for review]. An early example during this epoch of a simple model propounding the mediating role of cognition is the *stimulus-organism-response* model of William James' protégé, Robert Woodworth (19), but it was not until around the advent of what many consider the so-called “Cognitive Revolution” during the mid-20th century that more explicit cognitive-mediation models describing affective responding were formally developed. For example, clinical psychology has long appreciated the principle of cognitive mediation [see (20), for a review of this history], with Albert Ellis' (21, 22) *activating event-belief-consequence* (ABC) model of emotion. According this framework, emotional responses are largely the consequence of beliefs (e.g., appraisals) [see (23), for review], a particular class of propositional attitude (24), interacting with representations of goal-relevant events.

In cognitive psychology, theories of emotion generally accord to what has been termed the basic *modal model* of emotion, according to which emotional responses are part of a cyclic sequence: the *situation-attention-appraisal-response* sequence (25). For example, an individual attends to a goal-(in)congruent event and valuates (i.e., appraises) the descriptive representations of this fact in terms of its relevance to one's personal well-being (26), resulting in a valanced emotional response. This emotion-generative procedure is cyclical because responses are often the inputs to subsequent sequences; it is recursive and involves reciprocal causality (27–29). In clinical psychology, this is the framework on which all contemporary CBT-based forms of psychotherapy are predicated; that is, these theories postulate cognitive-vulnerability models [e.g., (30–35)]. More specifically, such interactions between the environment and cognition typically promote adaptive thoughts, feelings, and behaviors within the common range of human functioning, but they can also yield psychopathological symptoms. However, when the content of conceptual valuations (i.e., appraisals) lack empirical, logical, and practical grounding, they tend to engender maladaptive behavior and emotional distress whose intensity, frequency, and duration not only harm mental health and conflict with personal goals but also tend to self-reinforce and, therefore, develop in individuals a disposition toward similar patterns of thinking and responding in the future, leading to further goal obstruction (35). It is for these reasons, and sense of perniciousness, that such appraisals are referred to as irrational or dysfunctional in the literature.

## Cognitive Restructuring in Psychotherapy

So, cognition is contemporarily understood as a necessary mediator of the emotion-generative process, including those emotions which people would rather not experience and which detract from their mental health. Changing the dysfunctional aspects of cognition is typically taken on by the brains of other conspecifics in society, particularly clinicians, rather than on the part of the individual—at first. CBT-based schools of psychotherapy differ relative to one another on the nature of the dysfunctional cognitive operations underpinning particular types of mental illness and emotional distress and, therefore, also differ on the appropriate objects of cognitive change, but there are a number of rudimentary principles on which they agree (36). Namely, they differ from older schools of psychotherapy [e.g., psychoanalysis (37), person-centered therapy (38), etc.] in their de-emphasis of the past and adoption of an active-directive method of restructuring patients' thoughts [see (39), for a history of psychotherapy; see also (17, 40)]. More specifically, they accord to not only the principle of cognitive mediation but also that of cognitive penetrability (24): The idea that, in the case of human dysfunction, those cognitive operations mediating goal-incongruent events and unhealthy (negative or positive) emotions are modifiable. At a common-sense level of psychological explanation, this cognitive change is brought about via verbal intervention of a dialectical nature and, at a lower neurobiological level, by the same principles underpinning neurobiological change: activity-dependent, plasticity mechanisms [i.e., long-term potentiation; (41)]. Thus, cognitive restructuring refers to the aim and process of an intervention to supplant dysfunctional cognitions with more adaptive ones, and the predominate means by which to facilitate this cognitive change are verbal intervention strategies [see (42)]. However, as discussed below, bridging the explanatory gap between this understanding of cognitive change and one at the level of the brain and the information processing systems that work together to actuate it poses certain methodological challenges.

## Ecological Challenges to a Neuroscience of Cognitive Change

Cognitive neuroscientific research has discovered much in the clinical areas investigating relationships between the pathogenesis of psychopathological symptoms and abnormalities in the brain, such as hyper- and hypo-activation in regions supporting aspects of affective, semantic, and executive processing, as well as underconnectivity in the functional connections between such regions [see (43), for review]. Other research has focused on more structural, developmental, and genetic relations with functional aspects of the brain and psychopathological symptomology; a persisting problem in this enterprise that is worth noting is whether such brain abnormalities facilitate clinically significant symptoms or follow from them (44). As regards interventions and treatments for psychopathological symptoms, outcome measures of emotion, cognition, behavior, and physiology are best understood. These measures indicate the effects of an intervention. More successful treatments are those which lead to decreases in psychopathological symptoms and increases in positive emotion, rational beliefs, and adaptive behavior, as well as physiological changes in the brain (e.g., a less reactive amygdala in the case of anxiety disorders). However, little is understood at the levels of information processing and the brain about the cognitive and functional changes that take place throughout clinical interventions *in situ*—that underpin improvements to these features of mental health.

**Figure 1:**
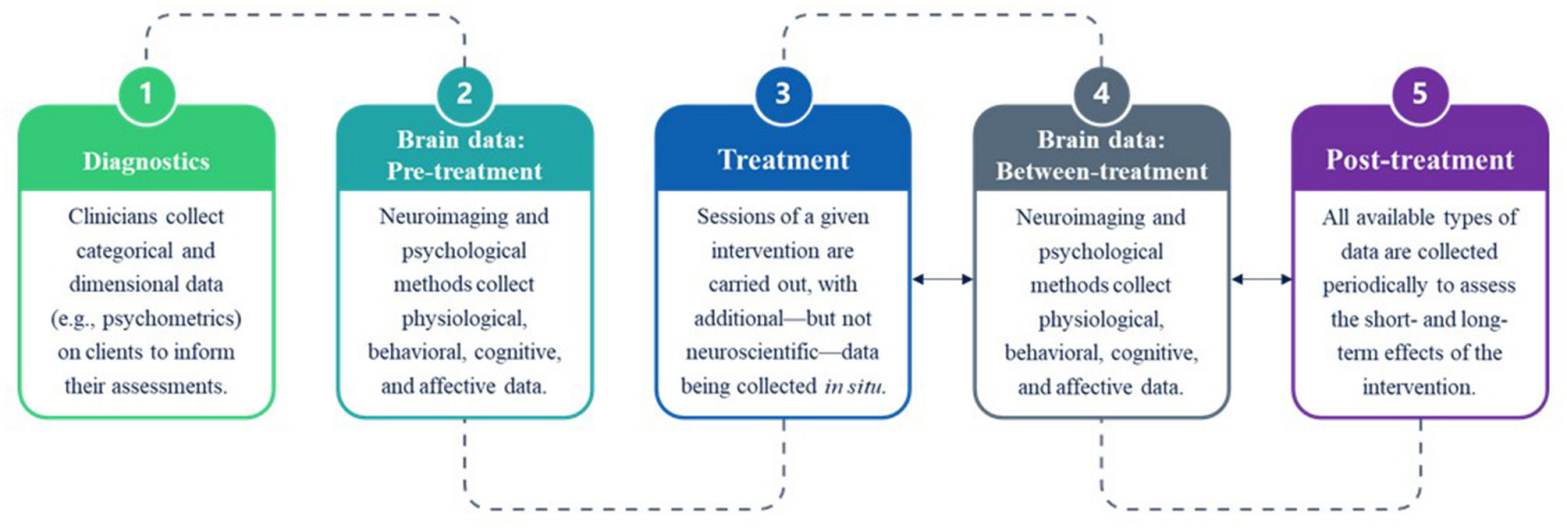
Data collection in the clinical neuroscience of mental health interventions. After an initial evaluation period (1), paradigms investigating the periodic effects of treatment (3) on the brain typically collect data from clinical populations before treatment commences (2), between particular stages of treatment (4), such as at 6 weeks, and at short- and long-term stages subsequent to treatment (5), such as after 14 weeks. Note that no neuroscientific data are collected during the interpersonal interactions driving the treatment sessions, leaving an explanatory gap regarding the nature of the information-processing systems in the brains of both clinicians and clients that contribute to the observed effects of interventions (e.g., cognitive change, emotion regulation, functional connectivity, etc.).

This is largely because experimental designs examining the effects of interventions on mental health only collect data periodically (e.g., before, halfway, and after treatment) rather than continuously within clinical settings at multiple periods [see (45)]. Methodologically, this has hitherto been infeasible, because neuroimaging techniques such as functional magnetic resonance imaging (fMRI) require clients to go to facilities to have their brains scanned. Such paradigms not only leave an explanatory gap regarding the neurocognitive mechanisms driving intervention effects but also raise a serious issue of ecological validity (Figure 1). The extent to which tasks represent functions at the level of the person and generalize in their predictability of responding in everyday-life situations is the degree to which they are valid, ecologically (46, 47). But when the neural correlates of cognitive change are investigated, studies typically use tasks relating to affective-reactivity, emotion regulation (e.g., reappraisal), or no task (e.g., resting-state) [e.g., (48–54)]. The blocked, event-related, or mixed experimental designs in these paradigms do not reflect the tasks of clients in clinical situations, nor does the testing environment reflect the interpersonal interactions (i.e., verbal intervention) that are integral to them. For example, although emotion regulation strategies for facilitating cognitive change have been found to correlate negatively with psychopathological symptoms [see (55), for review], these tasks fail to capture the recogitation that seems to be critical to disputing dysfunctional appraisal operations, do not target specific dysfunctional appraisal operations (i.e., instead, the contents and objects of reappraisal relate to the presented stimuli), and the stimuli seldom represent personally relevant, goal-incongruent situations; stimuli are also restricted to visual images rather than to the linguistic propositions clients might hear in real clinical situations. Thus, such paradigms are sufficient for investigating the neural bases of emotion regulation, but are ecologically limited in the ways they generalize to the clinical domain and, consequently, hinders our ability to bolster a neuroscientific understanding of the role of cognitive restructuring in cultivating mental health.

## Future Directions and Concluding Perspective

An important step toward addressing these ecological shortcomings and explanatory gaps in clinical cognitive neuroscience will be to take advantage of the ability of neuroimaging methods collect brain data in settings that better represent clinical situations. For example, functional near-infrared spectroscopy (fNIRS) is a relatively new, non-invasive neuroimaging technique (56, 57) that is similar to fMRI in its measurement of changes in concentrations of hemoglobin to infer changes in neural activation in the brain (i.e., neurovascular coupling), but differs in the sense that it is an optical rather than magnetic method: fNIRS calculates the attenuation of electromagnetic radiation, namely near-infrared light (650–1,000 nm), to index signal changes in oxygenated (HbO_2_) and deoxygenated (HbR) hemoglobin [see (58)]. For more in-depth discussions of the methodology of fNIRS and the quality control and publishing standards that are emerging in this field, see Orihuela-Espina et al. (59), Quaresima and Ferrari (60), and Yücel et al. (61). Recent technological advancements to the portability and wearability (62), as well as multi-person use (63), of fNIRS systems have broadened the types of experiments that can be carried out [see (64, 65), for reviews]. One of the most important applications of these advances has been to use fNIRS to investigate cognition in different environments outside of conventional laboratory settings [e.g., (66); see (67), for review]. For example, one study recently used a wireless system to examine the neural correlates supporting the balancing task of slacklining (68). It has been suggested that this improved ecological validity might also provide greater sensitivity and explanatory power to clinically-relevant subjects of interest (45). For example, investigating links between etiopathogenic mechanisms, cortical abnormalities (functional and structural), and psychopathological symptoms is crucial to better understanding mental *illness*, and fNIRS is playing an active part in this research enterprise [see (69–71), for reviews], as well as in similar ones in the clinical domain [see (72), for review]. While these areas of research are in line with what other neuroimaging methods are investigating, fNIRS is also uniquely well-suited to investigate how mental *health* is cultivated within clinical interventions.

**Figure 2:**
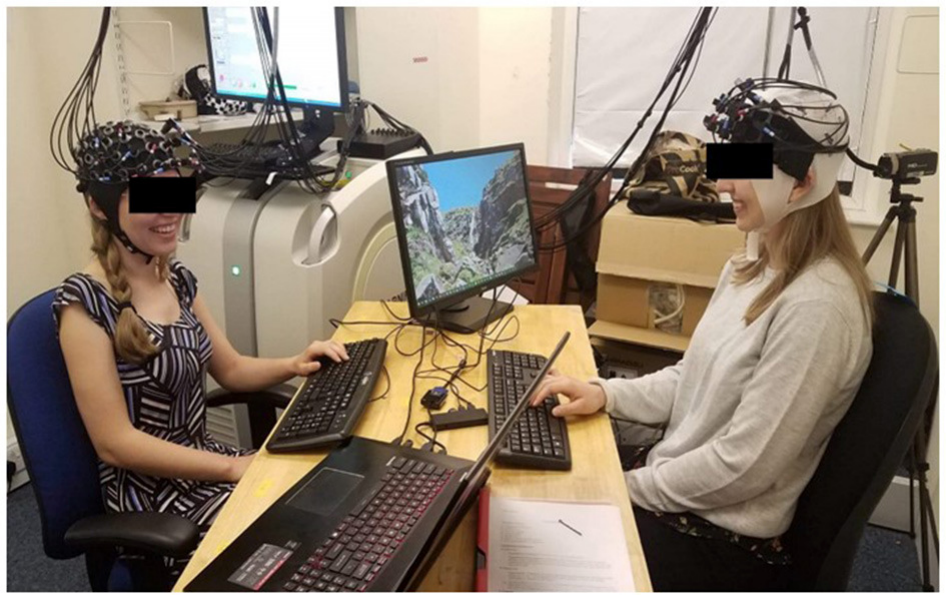
Example paradigm of a semi-clinical environment. Pairs of individuals are seated across a table with a full field of vision of each other in a normal room. A computer screen is positioned to the side of each participant's face to not obstruct natural facial information during verbal communication. Clinical interactions are then fractionated during fNIRS acquisition: A “client” utters a dysfunctional proposition while the “clinician” listens to this, then critically thinks about what makes the belief irrational, and finally verbally disputes the appraisal while the client carefully listens. These epochs would constitute one trial in a block; other blocks mnight involve reversing roles or changing the nature of the intervention strategy.

For example, multi-person fNIRS, namely fNIRS-based hyperscanning, is a technique by which hemodynamic changes and interpersonal brain dynamics (i.e., cross-brain synchronization) between two or more individuals can be observed whilst they engage in tasks in naturalistic or laboratory settings (73). This method would allow for the development of experimental designs that fractionate the interpersonal interactions occurring in real-world clinical settings and, importantly, to investigate the neurocognitive subsystems mediating cognitive restructuring, including those unique to clinicians. Hirsch et al. (74) represents a recent study that used fNIRS-based hyperscanning to examine the neural correlates of dialectical discourse. The design and multi-modal techniques employed in this study suggest a potentially promising direction for adapting future studies to use tasks involving verbal interactions that reflect the intervention strategies used in non-pharmacological treatments such as CBT-based interventions. For example, trials might be comprised of epochs for specific facets of verbal exchanges that typically occur between clinicians and clients (Figure 2). More specifically, one person (i.e., the client) utters some dysfunctional proposition and the other person (i.e., the clinician) listens to this, considers it, and then replies in a way that disputes it while the client listens. A design involving such epochs might require training prior to testing and some computer mediation (e.g., displaying propositions), at first, but would begin to bring a laboratory setting closer to a clinical one and, importantly, yield activation changes in the brain regions that are unique to each task and individual. Potentially, it could be those changes unique to the periods during which clinicians are required to think and speak in ways that dispute dysfunctional appraisals that become most critical to understanding how clients maintain their mental health subsequent to treatment, since such a paradigm raises the questions of how activation patterns between clinicians and clients on the same tasks might deviate and how these deviations might be experimentally rectified on the part of clients. In more naturalistic clinical situations, it might be difficult to separate these interpersonal interactions into clean epochs and blocks. Fortunately, data collected in ecological designs for which there is no *a priori* stimulus design and no computer mediation can now be analyzed. Namely, there are “brain-first” approaches to analyzing data to recover onsets and durations *a posteriori* [e.g., (75)]. Clearly, these possibilities suggest further research. Although there are other experimental methods capable of collecting data in intervention environments which might be useful in investigating these real-time interactions, such as electroencephalography, eye-gaze, ultrasound, heart rate, breathing rate, and forms of tomography, multi-modal approaches using a combination of these with fNIRS represent the best compromise of spatial and temporal resolution (76). In sum, assessing the intra- and inter-brain dynamics supporting the recogitation of thought in clinical situations might help to more clearly reveal the neurocognitive mechanisms underlying changes in mental health.

## Data Availability Statement

The original contributions presented in the study are included in the article/supplementary material, further inquiries can be directed to the corresponding author/s.

## Author Contributions

The author confirms being the sole contributor of this work and has approved it for publication.

## Conflict of Interest

The author declares that the research was conducted in the absence of any commercial or financial relationships that could be construed as a potential conflict of interest.
